# Correction to “Association between variation in hemoglobin A1c levels and diabetes therapy‐related quality of life in patients with diabetes”

**DOI:** 10.1111/jdi.14321

**Published:** 2024-09-27

Imai D, Ushigome E., Sakai R., Kitagawa N., Hamaguchi M., Yamazaki M., Fukui M. Association between variation in hemoglobin A1c levels and diabetes therapy‐related quality of life in patients with diabetes. J Diabetes Investig. 2024 Aug;15:1042–1046.

In the Data collection paragraph of the Method, VIM (variability independent of mean) is corrected to be MAD (mean absolute deviation). In addition, the calculation results of ARV were incorrect. The contents of Tables 1 and 2 have been partially revised accordingly.

We apologize for this error.

**Table 1:** Clinical characteristics of diabetes patients

Characteristics	
*N*	635
Male sex	51.1
Age (years)	67.0 (59.5–74.5)
Duration of diabetes mellitus (years)	11.0 (3.5–26.0)
Body mass index (kg/m^2^)	23.6 (20.8–26.3)
Blood glucose (mg/dL)	149 (119–184)
Blood glucose (SD) (mg/dL)	31.0 (17.0–47.8)
Hemoglobin A_1C_ (%)	7.3 (6.7–7.9)
Hemoglobin A_1C_ (mmol/mol)	55.9 (49.5–62.4)
Baseline Hemoglobin A_1C_ (%)	7.3(6.6–8.2)
Baseline Hemoglobin A_1C_ (mmol/mol)	55.9 (48.6–66.1)
Hemoglobin A_1C_ (SD) (%)	0.32 (0.12–0.52)
Hemoglobin A_1C_ (CV)	4.32 (1.71–6.83)
Hemoglobin A_1C_ (MAD)	0.25 (0.13–0.47)
Hemoglobin A_1C_ (ARV)	0.28 (0.16–0.48)
Number of HbA1c measurements (6/5/4)	84.9/8.5/6.6
Hypoglycemic drug use (including insulin)	73.1
Insulin use	36.9
Smoking (current/past)	9.0/20.0
Alcohol (everyday/social)	11.2/5.0
Nephropathy (normo‐/micro‐/macroalbuminuria)	69.8/23.8/6.4
Retinopathy (NDR/SDR/PDR)	85.7/8.7/5.6
Neuropathy	13.1
Macrovascular complication	6.5
Total DTR‐QOL score (%)	71.4 (59.8–83)
1. Burden on social activities and daily activities (%)	78.0 (64.8–91.2)
2. Anxiety and dissatisfaction with treatment (%)	66.1 (50.1–82.2)
3. Hypoglycemia (%)	78.5 (50.3–96.5)
4. Satisfaction with treatment (%)	60.7 (50.0–71.4)

For categorical variables, % is presented. For continuous variables, median (interquartile range) is presented.

ARV, average real variability; CV, coefficient of variation; DTR‐QOL, diabetes therapy‐related quality of life; MAD, mean absolute deviation; NDR, no diabetic retinopathy; PDR, proliferative diabetic retinopathy; SD, standard deviation; SDR, simple diabetic retinopathy.

**Table 2:** Univariate linear regression analyses on factors affecting total DTR‐QOL scores

Factors	Total DTR‐QOL scores	
	β	*P*
Age (years)	0.078	0.049
Sex (male = 1, female = 0)	−0.046	0.304
Duration of diabetes mellitus (years)	−0.154	<0.001
Body mass index (kg/m^2^)	0.099	0.013
Blood glucose (mg/dL)	0.021	0.069
Blood glucose (SD) (mg/dL)	−0.355	<0.001
Hemoglobin A_1C_ (mean) (%)	−0.271	<0.001
Hemoglobin A_1C_ (CV)	−0.157	<0.001
Hemoglobin A_1C_ (MAD)	−0.153	<0.001
Hemoglobin A_1C_ (ARV)	0.009	0.818
Retinopathy (no = 0, yes = 1)	−0.007	0.854
Nephropathy (no = 0, yes = 1)	0.058	0.144
Neuropathy (no = 0, yes = 1)	−0.019	0.633
Macrovascular complications (no = 0, yes = 1)	0.043	0.285
Insulin use (no = 0, yes = 1)	0.027	0.498

β indicates linear regression coefficient.

ARV, average real variability; CV, coefficient of variation; DTR‐QOL, diabetes therapy‐related quality of life; MAD, mean absolute deviation; SD, standard deviation.

